# Monthly Reports

**Published:** 1803-05-01

**Authors:** J. Ricards

**Affiliations:** 68, Hatton Street


### MONTHLY REPORTS.

*Cases admitted under the Care of the Surgeon of the Finsbury Dispensary, St. John's Square, Clerkenwell, from March* 10, *to April* 10, 1803.

| | | | |
|---|---|---|---|
| Phlegmone - - - - - | 1 | Tinea - - - - - - - | 1 |
| Herniæ Humoralis - - | 2 | Perniones - - - - - - | 2 |
| Abscessus - - - - - - | 7 | Ecchymoma - - - - - | 1 |
| Mortificationes - - - | 5 | Varrucæ - - - - - - | 2 |
| Caries - - - - - - - | 1 | Vagina imperforatu - - | 1 |
| Ulcera - - - - - - - | 10 | Fungus Funis Umbilicalis | 1 |
| Vulnera - - - - - - | 3 | Hernia - - - - - - - | 1 |
| Contusiones - - - - - | 6 | Hæmorrhois - - - - - | 1 |
| Spasma Malleoli - - - | 2 | Strictura - - - - - - | 1 |
| ——— Humeri - - - | 1 | Hematuria - - - - - - | 1 |
| Lues - - - - - - - - | 7 | Vacciola - - - - - - | 91 |
| Gonorrhœa - - - - - | 1 | | — |
| Prolapsus Ani - - - - | 1 | Total | 151 |
| Eruptio - - - - - - - | 1 | | — |

By the above list will be seen the great increase of the numbers of those who have been subjected to the Vaccine Inoculation at this Institution: it is scarcely necessary for me to add, that the most uniform success has attended every case: in some it has been necessary to repeat the operation of insertion, even although it is my constant practice (at the Dispensary) to inoculate with recent virus, by immediate transmission of it from one patient to another: this circumstance has been productive of some few days of delay, which has been the only inconvenience that has been in any case experienced; and in no one instance have I ever failed of ultimately producing the true pustule.

At the commencement of the practice of Vaccination much was said by different professional men, of extreme degrees of inflammation in the arms, and of ulceration succeeding to the desquamation of the scab of the pustule These circumstances were indeed considered by some persons as deducting considerably from the benignity of the disease; but of late we very seldom hear of an occurrence of this kind. I have myself had but one case of any soreness in the arm arising after the Vaccine Inoculation, which was during the last month, and which was occasioned by a fall which the child received, whereby the pustule was rubbed off by some gravel; the effect therefore could could not with propriety be attributed to the Vaccine dicease.

It cannot be doubted but that philosophy has contributed much to the art of healing, by the discovery of many very valuable and very powerful remedies; the most active of which perhaps is that of Electricity, a remedy than which there certainly is no one more decidedly useful, but whose real merits are not properly appreciated, from the neglect which it experiences from many practitioners, and from its too diffused and improper application by others; whereby, in the former case, its real agency in the cure of disease is not seen in those in which it might be servicable; and, on the other hand, by using it too indiscriminately, disgrace becomes attached to it from its frequent failure, because, in fact, it is not a cure for all diseases. I have been for some years past in the habit of employing this remedy in various diseases, and I may add, that in those cases where its application seemed to be indicated, it has been attended with great advantage.

I have lately had a case of Chorea Sancti Viti, which has received much benefit from electricity, and seems to be very quickly advancing to a state of perfect recovery. This patient is a child of eight years, who was sent to me by one of the Physicians of the Dispensary, for the benefit of electricity. She had been affected by this disease during several years, which existed in a very terrible degree, insomuch that every muscle was affected by it; the affection, beside the chronic state of the disease which existed, appeared to have violent paroxysms, or rather exacerbations, several times in the day, during which the spasms were so violent, that she became in a state resembling Epilepsy. The physician had suspected the presence of worms, and prescribed vermifuge medicines accordingly several times in large doses without success, and had, with different views, had recourse to various remedies. I passed some shocks through the body in different directions; she went home, and at the first evacuation afterwards discharged several large worms of the lumbrici kind; this circumstance did not surprise me; it was what I expected from what I had frequently found, that where worms had resisted the action of the most powerful internal medicines, a few shocks through the abdomen had caused their discharge. I have continued the use of Electricity to this child, and her disease has now nearly left her.

The

The astonishing powers of Galvanism, which I certainly consider as the same fluid identically with electricity, but acting under a particular modification, must, undoubtedly attract the attention of the medical world; the principal experiments which have been made on Galvanism have been philosophical and chemical; but when we consider its agency on the animal fibre, even for several hours after life has ceased, the difference of the shock from that of electricity, so much less violent to our sensations, yet so much more powerful in effect, insomuch, that a short continuance of almost imperceptible shocks shall be capable of producing very considerable vascular action on a part, and other very curious facts; we may, I think, deem it a subject worthy of inquiry, how it acts in disease. Little has yet been done on this subject in this country; but the reports of its wonderful success on the continent, induce us to hope, that in it we have the most powerful remedy hitherto known in many diseases; and in diseases, otherwise incurable. Some observations I have made, lead to the same conclusion; but as much investigation is requisite before we can adequately appreciate a new remedy, I have commenced a course of experimental inquiries upon this subject, in which I intend impartially to try its effects in various diseases, particularly deafness, paralysis, chronic rheumatism, and insanity, should cases occur; likewise various cases which come under the notice of the Surgeon; likewise its powers, which we have reason to expect to be great. in cases of suspended animation; and to draw a comparison between it and electricity in all cases. The results of these inquiries will form the subject of a separate publication.

